# Trends in Piezo Channel Research Over the Past Decade: A Bibliometric Analysis

**DOI:** 10.3389/fphar.2021.668714

**Published:** 2021-04-15

**Authors:** Jing Guo, Dongmei Gu, Tingting Zhao, Zhanhao Zhao, Yajun Xiong, Mengzhu Sun, Chen Xin, Yujie Zhang, Lixia Pei, Jianhua Sun

**Affiliations:** ^1^ Department of Acupuncture Rehabilitation, The Affiliated Hospital of Nanjing University of Chinese Medicine, Nanjing, China; ^2^ Department of Massage, Danyang Hospital of Traditional Chinese Medicine, Danyang, China; ^3^ School of Internet of Things Engineering, Jiangnan University, Wuxi, China; ^4^ Acupuncture and Massage College, Health and Rehabilitation College, Nanjing University of Chinese Medicine, Nanjing, China; ^5^ Acupuncture and Moxibustion Disease Project Group of China Evidence-Based Medicine Center of Traditional Chinese Medicine, Nanjing, China

**Keywords:** Piezo channels, bibliometric analysis, VOSviewer, web of science, co-citation analysis, co-occurrence analysis, co-authorship analysis

## Abstract

**Purpose:** We used bibliometric methods to evaluate the global scientific output of research on Piezo channels and explore the current status and trends in this field over the past decade.

**Methods:** Piezo channel-related studies published in 2010–2020 were retrieved from Web of Science. The R bibliometrix package was used for quantitative and qualitative analyses of publication outputs and author contributions. VOSviewer was used to construct networks based on co-authorship of countries/institutions/authors, co-citation analysis of journals/references, citation analysis of documents, and co-occurrence of keywords.

**Results:** In total, 556 related articles and reviews were included in the final analysis. The number of publications has increased substantially with time. The country and institution contributing the most to this field was the United States and Scripps Research Institute, respectively. Ardem Patapoutian was the most productive author and ranked first among the cited authors, *h*-index, and *m*-index. The top cited reference was the article published by Coste B et al. in *Science* (2010) that identified Piezo1/2 in mammalian cells. The top journals in terms of the number of selected articles and citations were *Nature Communications* and *Nature*, respectively. The co-occurrence analysis revealed that Piezo channels are involved a variety of cell types (Merkel cells, neurons, endothelial cells, red blood cells), physiological processes (touch sensation, blood pressure, proprioception, vascular development), related ion channels (transient receptor potential, Gardos), and diseases (pain, distal arthrogryposis, dehydrated hereditary stomatocytosis, cancer), and pharmacology (Yoda1, GsMTx-4).

**Conclusion:** Our bibliometric analysis shows that Piezo channel research continues to be a hotspot. The focus has evolved from Piezo identification to architecture, activation mechanism, roles in diseases, and pharmacology.

## Introduction

Mechanotransduction, referring to the conversion of mechanical forces into electrochemical signals, plays a critical role in various forms of physiological or pathophysiological processes in mammalian cells, including touch, proprioception, hearing, pain, vascular development, and blood pressure regulation (Chalfie, 2009; Tsunozaki and Bautista, 2009). Mechanosensitive (MS) ion channels are functionally conserved from prokaryotes to eukaryotes. MS ion channel activity has been detected in nearly every organism, and is essential to the life (Gillespie and Walker, 2001). MS channels specialized in mechanotransduction are directly activated by stresses to the lipid bilayer or its associated nonmembrane components (Jin et al., 2020). Several MS ion channels have been identified according to the criteria proposed by Arnadóttir et al. (2011), and include MS channels of large/small conductance (MscL/MscS), degenerin/epithelial sodium channel (DEG/ENaC) channels, TREK/TRAAK channels, transient receptor potential (TRP) channels, TMC1/2 channels, and Piezo channels (Jin et al., 2020).

Two force transduction and gating models have been proposed to understand the gating mechanism of MS channels: the membrane tension model and the tether model (Jin et al., 2020). The membrane tension model proposes that the gating of the channel comes from the change in lipid bilayer tension (Martinac et al., 1990), such as MscL/MscS (Sukharev et al., 1993), TREK/TRAAK channels (Sukharev et al., 1993; Brohawn et al., 2014a; Brohawn et al., 2014b), OSCA channels (Murthy et al., 2018a), and Piezo1 (Cox et al., 2016). In the tether model, force is transmitted through a tether connecting the channel with ectomembrane components (molecules from the extracellular matrix [ECM] or intracellular cytoskeleton) to gate the channel. The NOMPC channels in *Drosophila* and the DEG/ENaC channels in nematodes are well-investigated examples of the tether model (Emtage et al., 2004; Arnadóttir et al., 2011; Zhang et al., 2015). Although considerable progress in the study of the K2P channel family in mammals has been made, the MS ion channels in mammals that depolarize cells remained unclear because activation of K2P channel is hyperpolarization. On the other hand, it has been controversially proposed that, for the TRP channel family, mechanical stimuli may not directly gate the ion channels by force, but instead may trigger second messenger signaling to activate downstream ion channels (Christensen and Corey, 2007; Gottlieb et al., 2008; Patel et al., 2010; Nikolaev et al., 2019). In this case, the ion channels are mechanosensitive but are not mechanically gated. Nevertheless, it is generally believed that the common forms of mechanical sensation are mediated by MS channels that are directly gated by force (Gillespie and Walker, 2001). The molecular identities of the MS channels in mammals largely remained elusive.

**FIGURE 1:**
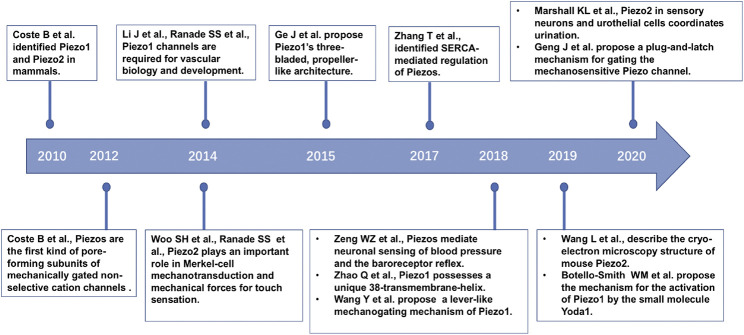
The timeline of part of the key discoveries in Piezo channel research.

The discovery of the Piezo channel family, which shed light on this enigma, paved the way for understanding the discriminative molecular identities of mechanotransduction for sensory signals (Bagriantsev et al., 2014). In 2010, Patapoutian and colleagues demonstrated that Piezo1 and Piezo2 proteins, encoded by the *PIEZO1*/*FAM38A* and *PIEZO2*/*FAM38B* genes, respectively, and identified as mechanically activated (MA) cation channels, were crucial required in mammalian cells (Coste et al., 2010). Overexpression of mouse Piezo1 or Piezo2 induced two different dynamic MA currents (Coste et al., 2010). Subsequently, a series of studies on Piezo protein architecture (Coste et al., 2012; Ge et al., 2015; Guo and MacKinnon, 2017; Saotome et al., 2018; Zhao et al., 2018; Wang et al., 2019), Piezo distribution in mammalian cells (Li et al., 2014; Woo et al., 2014), Piezo function in physiology and associated disease states (Ranade et al., 2014a; Ranade et al., 2014b; Woo et al., 2014; Woo et al., 2015; Marshall et al., 2020), and molecular mechanisms (Zhang et al., 2017) were established (Figure 1). Piezo1 and Piezo2 are large membrane proteins that contain over 2,500 and 2,800 amino acid residues, respectively, and show little homology to other known ion channels. Piezo channels are the first established family of non-selective MS cation channels in mammals. Piezo proteins subjected to mechanical force, comprising compression, tension, swelling, and shear stress, release cations and then induce cell excitation and signal transmission. Piezo1 is involved in various key biological activities, including vascular and lymphatic development (Ranade et al., 2014a; Li et al., 2014; Nonomura et al., 2018), regulating blood pressure (Wang et al., 2016; Zeng et al., 2018), regulating red blood cell (RBC) volume (Cahalan et al., 2015), the perception of bladder endothelial cells and renal tubular epithelial cells to force (Peyronnet et al., 2013; Miyamoto et al., 2014), and bone formation (Sun et al., 2019). Piezo2 mediates mechanotransduction process in the somatosensation of touch (Ranade et al., 2014b; Woo et al., 2014), proprioception (Woo et al., 2015), and pain (Eijkelkamp et al., 2013; Ferrari et al., 2015; Murthy et al., 2018b) in mammals. Mutations in human *PIEZO1* and *PIEZO2* can lead to a variety of hereditary diseases, including Piezo1-based dehydrated hereditary stomatocytosis (DHS) (Zarychanski et al., 2012) and generalized lymphatic dysplasia (Lukacs et al., 2015), and Piezo2-based distal arthrogryposis syndrome (Coste et al., 2013; McMillin et al., 2014), Gordon syndrome and Marden-Walker syndrome (McMillin et al., 2014).

Bibliometrics is the quantitative and qualitative analysis of published academic literature for tracking the development of a certain research field over a defined time frame. It focuses on the impacts of publications, contributions of individuals/institutes/countries, patterns of authorship, and the main direction of future research in the field. Considering that studies on Piezo channels began in 2010 and have developed rapidly over the past ten years, it is necessary to explore how the research has changed over time and to predict future trends. However, no prior bibliometric analyses of the global literature on Piezo channels have been conducted to date. In the present study, we conducted a bibliometric analysis of studies on Piezo channels published over the past decade at a global level to illustrate the research landscape and explore the hot topics and emerging trends.

## Methods

### Data Source and Search Strategy

All data were retrieved from the Web of Science (WoS) Core Collection including SCI-EXPANDED on January 26, 2021. The search terms were “Piezo”, “Piezos”, “Piezo1”, “Piezo2”, “DmPiezo”, “FAM38A”, and “FAM38B″ with publication timespan (2010–2020). The document types were restricted to articles or reviews (*n* = 5057). Publications that were unrelated to the search topic (*n* = 4501) were excluded. The final analysis contained 556 records that were downloaded as a. txt file from the WoS.

### Bibliometric Analysis and Visualization

The bibliographic information of the selected publications was converted and analyzed automatically using the bibliometrix package in R 4.0.3, and included the distribution of countries/regions, years of publication, and authors. Publication quality by author was assessed based upon metrics that included the number of publications, citations in the research area, publication *h*-index value, and *m*-index value. The *h*-index is used to quantify an individual’s scientific research output and measure his citation impact (Hirsch, 2005). The *m*-index was proposed to facilitate comparisons between academics with different academic careers lengths : $m_{index}=\frac{h_{index}}{Y_{academic\;age}}$ (*Y*_academic age_ is measured as the number of years since the first published paper in the research area) (Hirsch, 2005). The journal impact factors used for individual publications were collected from the 2019 Journal Citation Rreports (JCR) (Clarivate Analytics, Philadelphia, United States).

Networks were constructed using VOSviewer (Version 1.6.16, Leiden University, the Netherlands) (van Eck and Waltman, 2010; 2017): co-authorship analysis of countries/institutions/authors, co-citation analysis of journals/references, citation analysis of documents, and co-occurrence analysis of keywords. Further, keywords that occurred more than five times were presented in three visualizations (network, overlay, density visualization) of the co-occurrence analysis to identify important terms in Piezo channel research.

## Results

### The Trends in Global Publications

**FIGURE 2:**
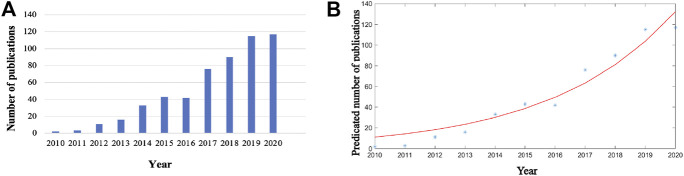
Global trends in publications on Piezo channels research. **(A)** Single-year publication output over the past decade. **(B)** Model fitting curves of growth trends in publications.

A total of 556 articles from 2010 to 2020 and related to Piezo channels were retrieved from WoS. From two articles (0.35%) in 2010 to 117 articles (21.04%) in 2020, global publications in the field exhibited a strong growth trend (Figure 2A). The time curve constructed by the logistic regression model suggested that the field is currently in a phase of steady growth in global publication output (Figure 2B).

### Distribution of Countries and Institutions

**FIGURE 3:**
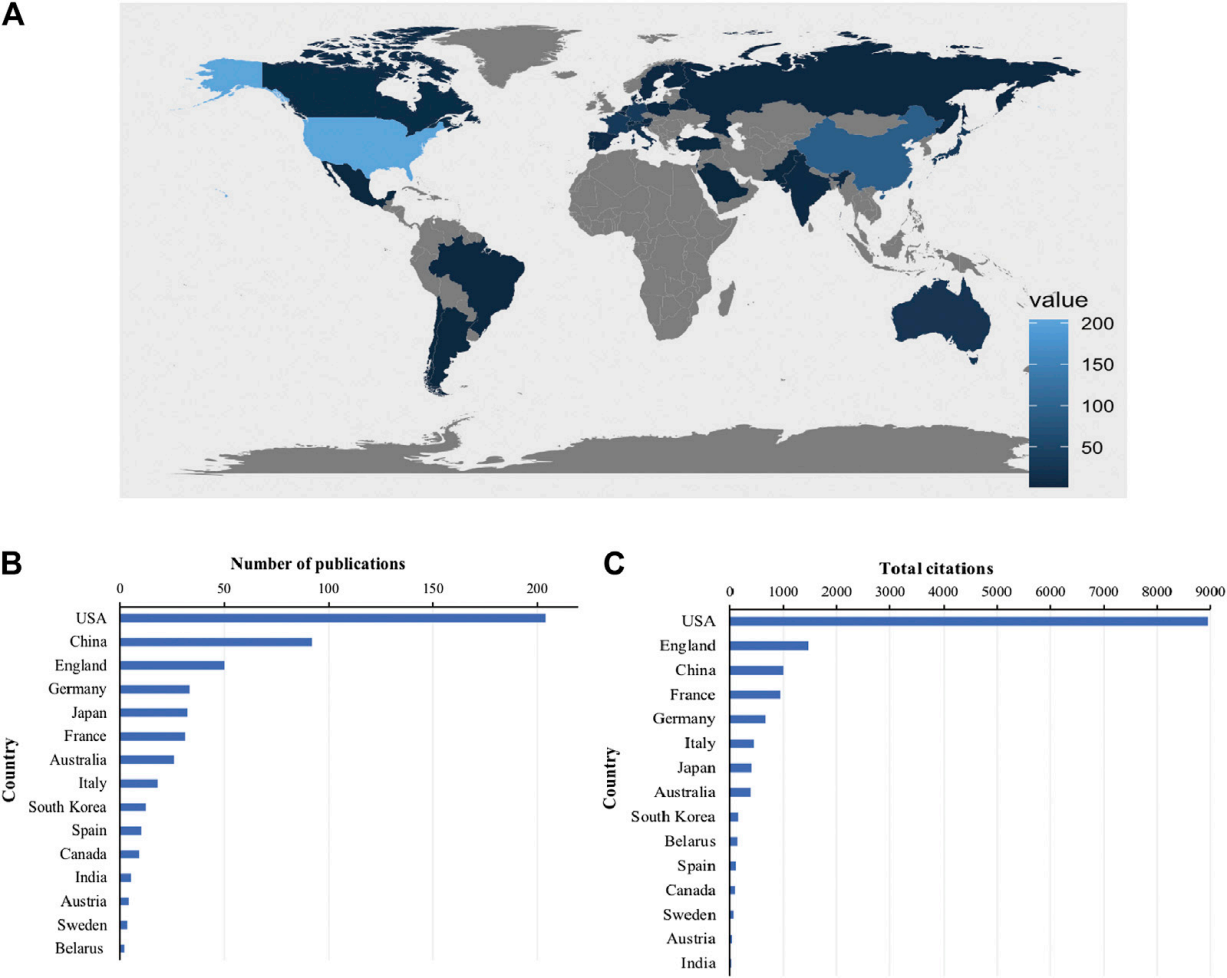
Countries contributing to Piezo channels research. **(A)** World map showing the distribution of countries in this field. **(B)** Top 15 countries with the largest number of publications. **(C)** Total citations of related articles from different countries.

Global contributions to Piezo channel research were analyzed and represented by R in a blue-coded world map (Figure 3A)*.* A total of 53 countries and regions contributed to publications in this field. The United States contributed the greatest number of articles (204, 36.69% of all articles), followed by China (92, 16.55%), England (50, 8.99%), Germany (33, 5.94%), and Japan (32, 5.76%) (Figure 3B). Studies from the United States had the highest number of citations (8954 citations), followed by those from England (1467 citations), China (997 citations), France (930 citations), and Germany (663 citations) (Figure 3C).

**FIGURE 4:**
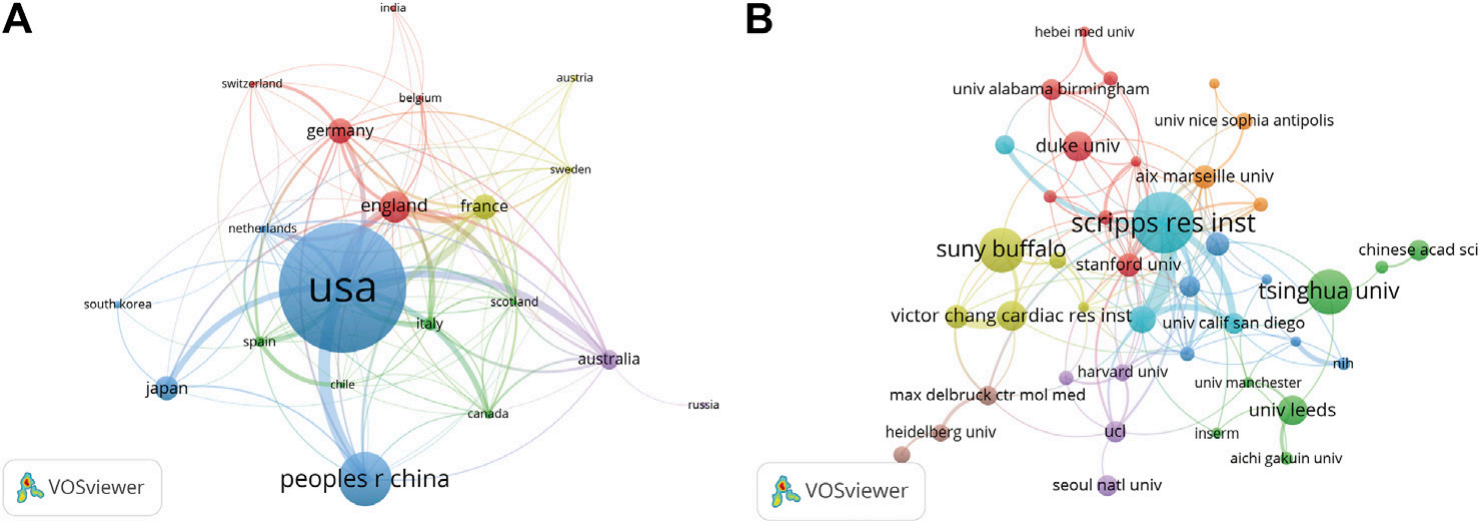
Co-authorship analysis of countries and institutions. **(A)** Network map of co-authorship between countries with more than five publications. **(B)** Network map of co-authorship between institutions with more than five publications. The thickness of the lines indicates the strength of the relationship.

A total of 20 countries with more than five publications in the field were analyzed in the co-authorship analysis (Figure 4A). The five countries with the highest total link strength were the United States (total link strength = 135 times), England (81), France (61), Germany (54), and Australia (48).

A total of 821 institutions were involved in this field. Scripps Research Institute (29 records, 5.22% of all articles) contributed the most publications, followed by State University of New York (SUNY) at Buffalo (21, 3.78%), Tsinghua University (21, 3.78%), Duke University (14, 2.52%), and University of Leeds (14, 2.52%).

We analyzed the co-authorship of 50 institutions with more than five publications. The exclusion of nine items that were not connected revealed the collaborations of 41 institutions (Figure 4B). The five institutions with the highest total link strength were Scripps Research Institute (total link strength = 54 times), Novartis (33), Stanford University (21), Aix-Marseille University (17), and University of Pennsylvania (17).

### Analysis of Journals and Research Areas

A total of 556 articles were published in 252 journals. Table 1 shows the top 10 most popular journals for publishing articles on Piezo channels. *Nature Communications* (26 records, 4.68% of all articles) had the most publications, followed by *Proceedings of the National Academy of Sciences of the United States of America* (*PNAS*) (24, 4.32%), *Scientific Reports* (23, 4.14%), *Cell Reports* (21, 3.78%), and *eLife* (19, 3.42%).

**TABLE 1:** Top 10 popular journals and cited journals.

Rank	Popular journals	Records (n)	2019 impact factor	2019 JCR partition	Ccited journals	Citations (n)	2019 impact factor	2019 JCR partition
1	Nat commun	26	12.121	Q1	Nature	2287	42.778	Q1
2	Proc Natl Acad Sci United States	24	9.412	Q1	Proc Natl Acad Sci United States	1456	9.412	Q1
3	Sci rep	23	3.998	Q1	Nat Commun	927	12.121	Q1
4	Cell rep	21	8.109	Q1	Science	867	41.846	Q1
5	eLife	19	7.08	Q1	Neuron	667	14.415	Q1
6	Nature	18	42.778	Q1	J Biol Chem	656	4.238	Q2
7	Curr top membr	12	3.744	Q3	eLife	602	7.08	Q1
8	PLoS one	12	2.74	Q1	Blood	599	17.543	Q1
9	Neuron	11	14.415	Q1	Cell	574	38.637	Q1
10	Front physiol	10	3.367	Q1	J Physiol-London	542	4.547	Q1

**FIGURE 5:**
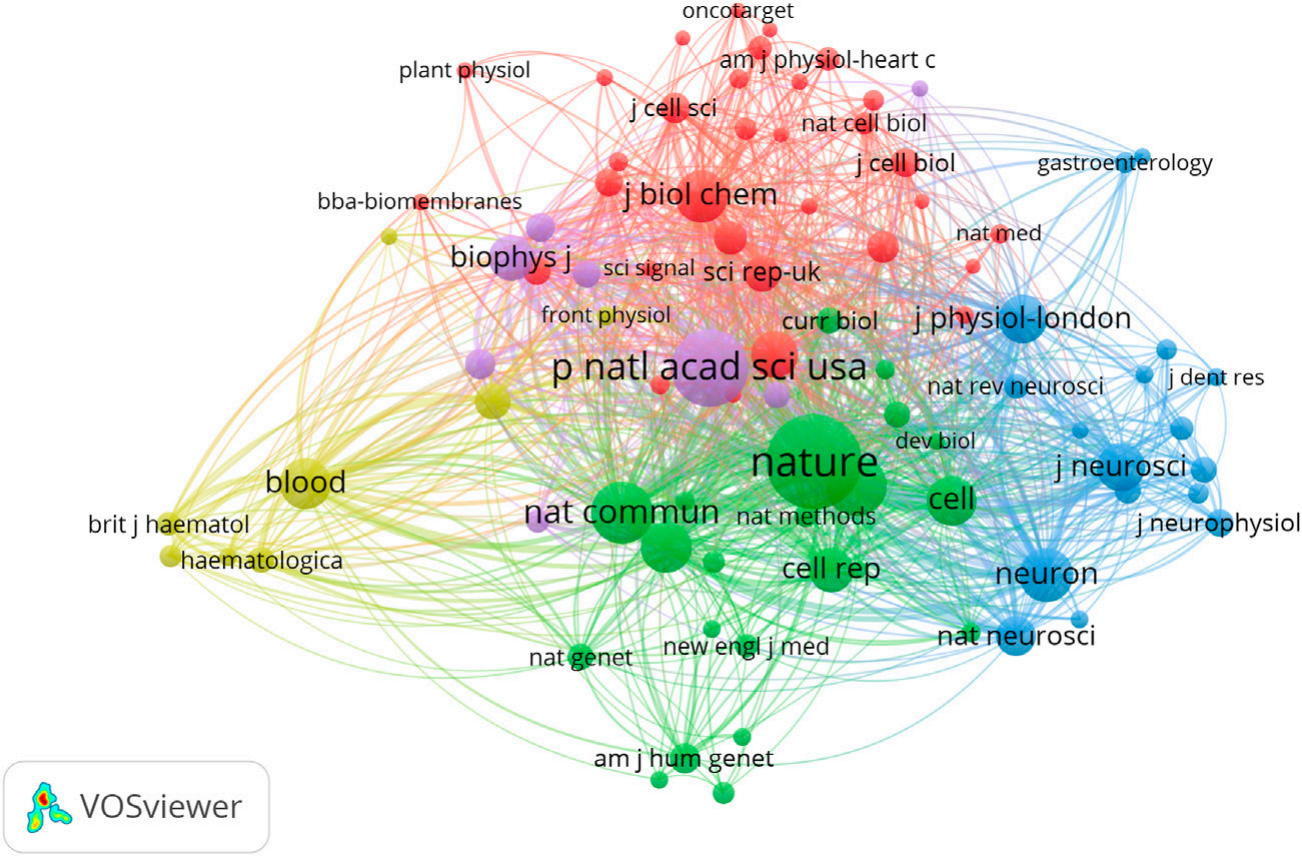
Network map of journals that were co-cited in more than 50 publications.

We analyzed a total of 87 journals for all publications that were co-cited in more than 50 publications (Figure 5). Table 1 shows the top ten cited journals that published related articles. *Nature* had the largest number of citations (2287 citations), followed by *PNAS* (1456 citations), *Nature Communications* (927 citations), *Science* (867 citations), and *Neuron* (667 citations).

In total, identified publications were classified under 52 research areas. The most well-represented research area was Science and Technologies (76 records, 13.67% of all articles), followed by Multidisciplinary Sciences (70, 12.59%), Biochemistry and Molecular Biology (61, 10.97%), Biochemistry and Neurology (50, 8.99%), and Neurosciences (47, 8.45%) (Table 2 **)**.

**TABLE 2:** Top ten well-represented research areas.

Rank	Research areas	Records (n)	% (of 556)
1	Science and technology	76	13.67
2	Multidisciplinary sciences	70	12.59
3	Biochemistry and molecular biology	61	10.97
4	Biochemistry and neurology	50	8.99
5	Neurosciences	47	8.45
6	Cell biology	45	8.09
7	Biophysics	25	4.50
8	Physiology	22	3.96
9	Hematology	19	3.42
10	Research and experimental medicine	14	2.52

### Analysis of Authors

**FIGURE 6:**
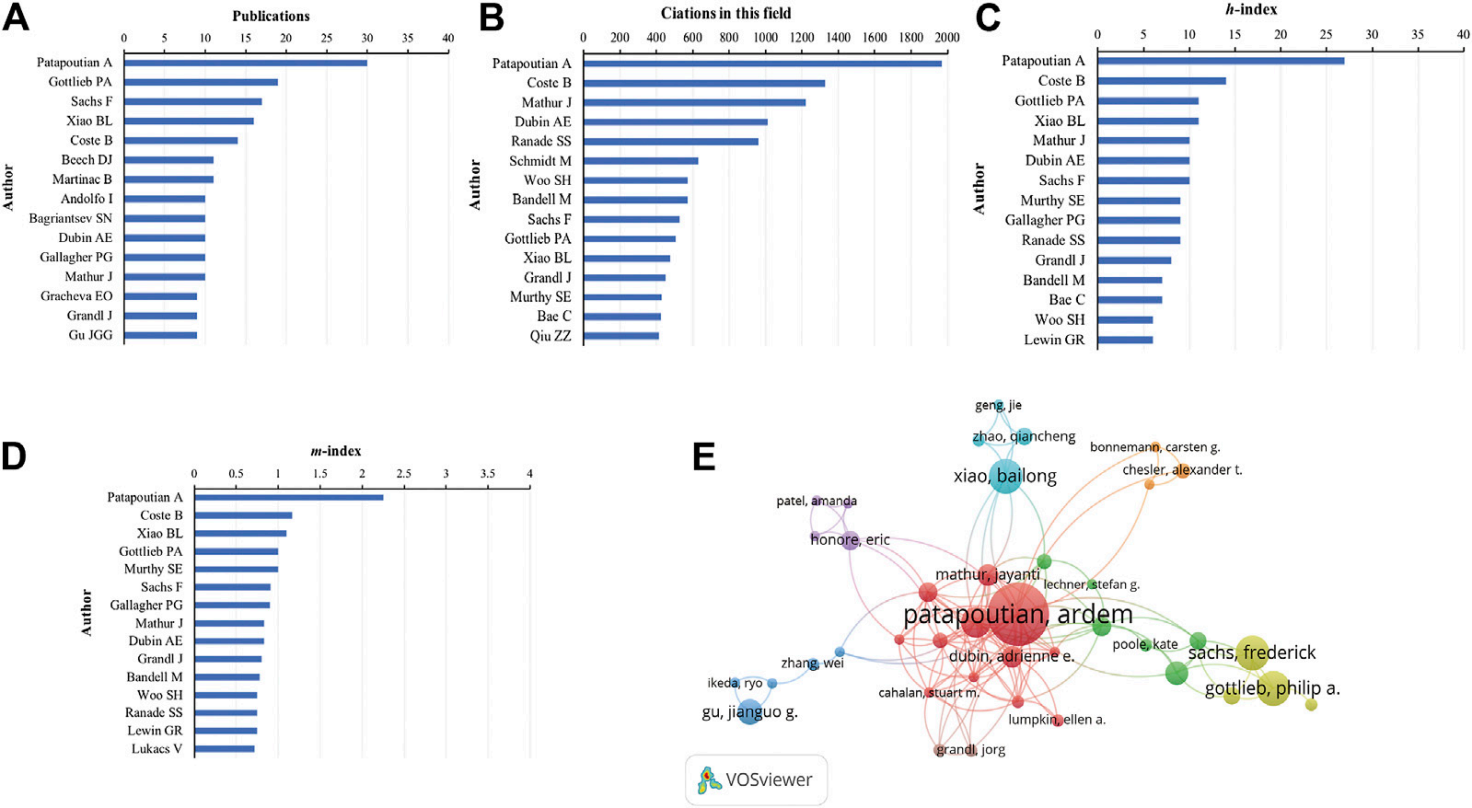
Analysis of authors. **(A)** Number of publications from different authors. **(B)** Total citations in the research filed from different authors. **(C)** *h*-index of publications from different authors. **(D)** *m*-index of publications from different authors. **(E)** Network map of co-authorship between authors with more than five publications.

In terms of the number of publications, Patapoutian A. was the most productive author, with 30 articles (5.40% of all articles), followed by Gottlieb P. (19, 3.42%), Sachs F. (17, 3.06%), Xiao B. (16, 2.88%), and Coste B. (14, 2.52%) (Figure 6A). In terms of citations in this field, Patapoutian A. was ranked first (1969 citations), followed by Coste B. (1327 citations), Mathur J. (1220 citations), Dubin A.E. (1013 citations), and Ranade S.S. (959 citations) (Figure 6B).

Publications from Patapoutian A. had the highest *h*-index (27), followed by that from Coste B. (14), Gottlieb P. (11), Xiao B. (11), and Mathur J. (10) (Figure 6C). The *m*-index of publications from Patapoutian A. (2.25) was also ranked first, followed by that from Coste B. (1.17), Xiao B. (1.10), Gottlieb P. (1.00), and Murthy S.E. (1.00) (Figure 6D).

We analyzed a total of 62 authors that were co-authored in more than five publications. The exclusion of 12 items that were not connected revealed the collaborations of 40 authors (Figure 6E). The five authors with the highest total link strength were Patapoutian A. (total link strength = 91 times), Coste B. (54), Mathur J. (48), Dubin A.E. (41), and Bandell M. (31).

### Citation and Co-Citation Analyses

**FIGURE 7:**
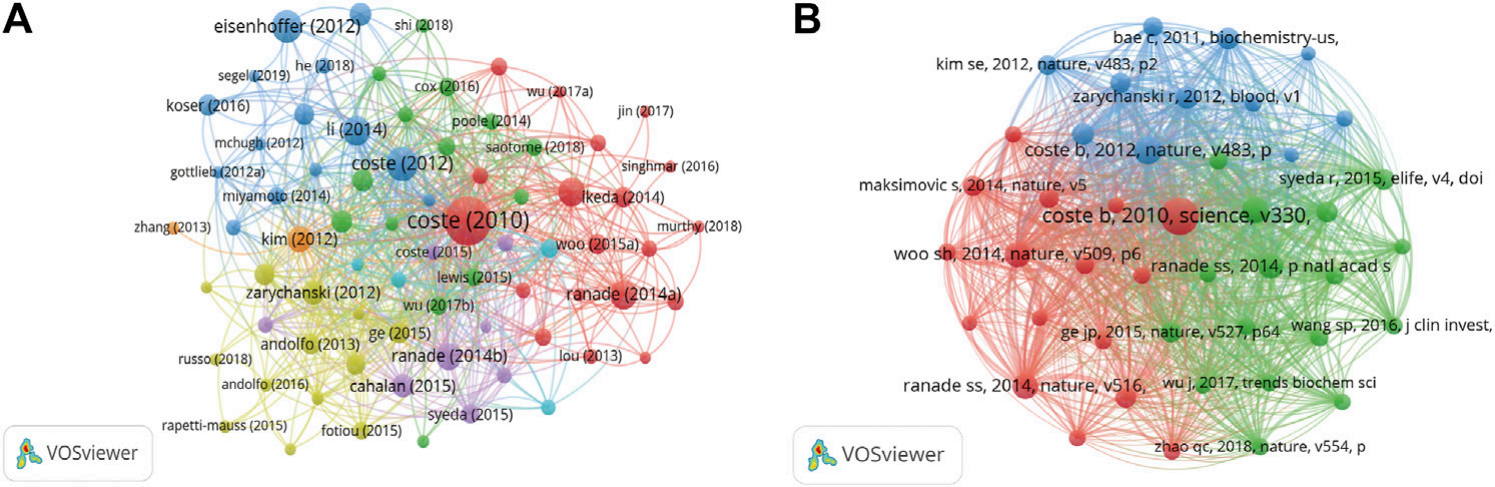
**(A)** Network map of citation analysis of documents with more than 50 citations. **(B)** Network map of co-citation analysis of references with more than 50 citations.

The citation analysis showed that 74 documents had more than 50 citations (Figure 7A). Table 3 lists the top ten documents with the highest citations. There were 819 citations for “Piezo1 and Piezo2 are essential components of distinct mechanically activated cation channels” (Coste et al., 2010), followed by “Piezo proteins are pore-forming subunits of mechanically activated channels” (Coste et al., 2012), with 413 citations. The third-ranked article for the largest number of citations was “Crowding induces live cell extrusion to maintain homeostatic cell numbers in epithelia” (Eisenhoffer et al., 2012), with 354 citations.

**TABLE 3:** Top ten citation analysis of documents on Piezo channel research.

Rank	Title	First author	Source	Publication year	Citations (n)
1	Piezo1 and Piezo2 are essential components of distinct mechanically activated cation channels	Coste et al. (2010)	*Science*	2010	819
2	Piezo proteins are pore-forming subunits of mechanically activated channels	Coste et al. (2012)	*Nature*	2012	413
3	Crowding induces live cell extrusion to maintain homeostatic cell numbers in epithelia	Eisenhoffer et al. (2012)	*Nature*	2012	354
4	Piezo1 integration of vascular architecture with physiological force	Li et al. (2014)	*Nature*	2014	308
5	Piezo2 is required for merkel-cell mechanotransduction	Woo et al. (2014)	*Nature*	2014	283
6	Piezo2 is the major transducer of mechanical forces for touch sensation in mice	Ranade et al. (2014b)	*Nature*	2014	277
7	Piezo1, a mechanically activated ion channel, is required for vascular development in mice	Ranade et al. (2014a)	*Proc Natl Acad Sci USA*	2014	251
8	The role of drosophila piezo in mechanical nociception	Kim et al. (2012)	*Nature*	2012	228
9	Mutations in the mechanotransduction protein PIEZO1 are associated with hereditary xerocytosis	Zarychanski et al. (2012)	*Blood*	2012	200
10	Mechanical stretch triggers rapid epithelial cell division through Piezo1	Gudipaty et al. (2017)	*Nature*	2017	182

We analyzed 43 references that were co-cited in more than 50 citations (Figure 7B). Table 4 lists the top ten references with the highest citations. The five references with the largest number of citations were by Coste B. (2010, *Science*; 363 citations), Coste B, (2012, *Nature*; 193 citations), Li J. (2014, *Nature*; 171 citations), Ranade S.S., (2014, *Nature*; 160 citations), and Woo S.H. (2014, *Nature*; 160 citations).

**TABLE 4:** Top ten co-citation analysis of cited reference on piezo channels research.

Rank	Title	First author	Source	Publication year	Citations (n)
1	Piezo1 and Piezo2 are essential components of distinct mechanically activated cation channels	Coste et al. (2010)	*Science*	2010	363
2	Piezo proteins are pore-forming subunits of mechanically activated channels	Coste et al. (2012)	*Nature*	2012	193
3	Piezo1 integration of vascular architecture with physiological force	Li et al. (2014)	*Nature*	2014	171
4	Piezo2 is the major transducer of mechanical forces for touch sensation in mice	Ranade et al. (2014b)	*Nature*	2014	160
5	Piezo2 is required for merkel-cell mechanotransduction	Woo et al. (2014)	*Nature*	2014	160
6	Piezo1, a mechanically activated ion channel, is required for vascular development in mice	Ranade et al. (2014a)	*Proc Natl Acad Sci USA*	2014	150
7	Mutations in the mechanotransduction protein Piezo1 are associated with hereditary xerocytosis	Zarychanski et al. (2012)	*Blood*	2012	119
8	The mechanosensitive ion channel Piezo1 is inhibited by the peptide GsMTx4	Bae et al. (2011)	*Biochemistry*	2011	113
9	Xerocytosis is caused by mutations that alter the kinetics of the mechanosensitive channel Piezo1	Bae et al. (2013)	*Proc Natl Acad Sci USA*	2013	111
10	Piezo1 links mechanical forces to red blood cell volume	Cahalan et al. (2015)	*eLife*	2015	107

### Co-Occurrence Analysis of Keywords

**FIGURE 8:**
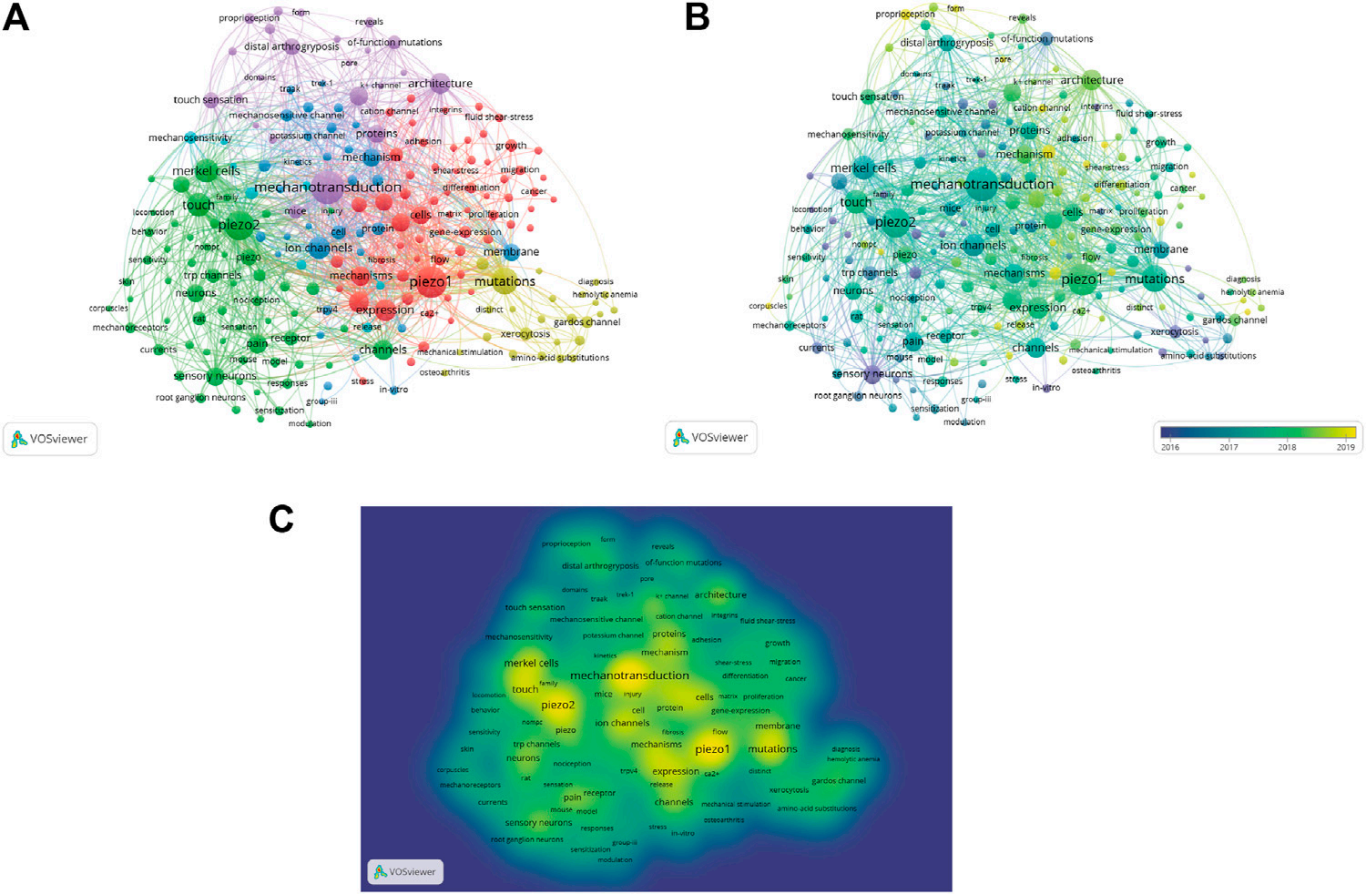
Co-occurrence analysis of keywords. **(A)** Mapping of keywords of studies. **(B)** Distribution of keywords according to average publication year (blue: earlier, yellow: later). **(C)** Distribution of keywords according to the mean frequency of appearance. Keywords in yellow occurred with the highest frequency.

We analyzed a total of 212 keywords that were identified as having occurred more than five times (Figure 8A). The colors in the overlay visualization shown in Figure 8B indicate the average publication year of the identified keywords. The majority of the keywords were published after 2017, with greener or yellower colors. The density visualization showed the same identified keywords mapped by frequency of appearance (Figure 8C).

## Discussion

### General Trends in the Piezo Channels Research

In the present study, we combined bibliometric analyses with network visualizations to characterize the current landscape of the Piezo channel research, analyzing the contributions of countries, institutions, journals, and authors to this emerging field, and predicting hot topics that will be of continued research interest in the coming years. Since the field emerged in 2000, the annual publication output in the field has increased steadily, with particularly rapid increases seen over the past ten years, accounting for 41.73% of all of the identified articles.

With the largest number of publications and citations, and the top rank for co-authorship analysis by country, the United States is currently the world leader in Piezo channel research. These results suggest that the United States may have a significant impact on the direction of research in this field and encompasses the strongest collaborations worldwide. The number of studies conducted in China, England, Germany, and Japan has also risen substantially during the past decade, accounting for 37.24% of all of the included studies. China was ranked second in the total number of publications, and third in the total citations; it was ranked sixth in collaboration with other countries. Scripps Research Institute was the most productive and was ranked first in co-authorship analyses conducted by institution, suggesting its close cooperation with other institutions.

### Influential Authors and Studies in the Piezo Channels Research

Patapoutian A. of Scripps Research Institute is pioneered in the field of Piezo channels, and has the largest number of publications and citations, and the top rank for *h*-index, *m*-index, and co-authorship analysis conducted by author. He has an *m*-index of 2.25, suggesting that he can be characterized as an outstanding scientist, likely to be found only at the top universities or major research laboratories (Hirsch, 2005). Dr. Patapoutian is interested in ion channels and other cellular sensors that convert mechanical stimuli into chemical signals (Peier et al., 2002a; Peier et al., 2002b; Story et al., 2003; Moqrich et al., 2005; Syeda et al., 2016b). As the role of DEG/ENaC and TRP channels in mammalian mechanotransduction remains controversial (Christensen and Corey, 2007; Gottlieb et al., 2008; Nikolaev et al., 2019), the identification of novel ion channel families is critical for further understanding of mammalian mechanotransduction (Ranade et al., 2015). Patapoutian and colleagues identified a novel MA cation channel family, named Piezo 1 and Piezo 2, which are expressed in many MS cell types, such as RBCs and vascular endothelial cells (Ranade et al., 2014a; Cahalan et al., 2015), and in touch and proprioceptive neurons (Ranade et al., 2014b; Woo et al., 2014). His team’s current efforts focus on understanding the structure-function relationship of Piezo proteins and elucidating their physiological roles in biological processes and diseases involved in mechanotransduction (Murthy et al., 2018b; Nonomura et al., 2018; Zeng et al., 2018; Marshall et al., 2020).

Xiao B. from Tsinghua University, China, underwent his postdoctoral training with Dr. Patapoutian from 2007 to 2012 and contributed to the identification and characterization of several classes of ion channels, including Piezo channels (Coste et al., 2012). His team currently focuses on solving the scientific question of how mechanically gated Piezo channels convert mechanical stimulation into electrochemical signals at the molecular, cellular, and organism level. He has made a series of important research achievements in the three-dimensional (3D) structure analysis of mechanically gated Piezo channels (Ge et al., 2015; Zhao et al., 2019), the identification and discovery of key functional sites, the revelation of ion permeability and mechano-gating mechanism (Wang et al., 2018; Zhao et al., 2018), the regulation of protein interaction (Zhang et al., 2017), and the discovery of small-molecular drugs (Xiao, 2020).

The citation analysis of documents and co-cited analysis of references showed that Coste et al. (2010, *Science*), with the highest number of citations, identified a path in Piezo channel research. Piezo1, encoded by *FAM38A*, was identified using small interfering RNA (siRNA) knockdown screening in the mouse Neuro2A neuroblastoma cell line. Piezo1 is essential for mediating the endogenous MA currents. Homologous Piezo2 was subsequently discovered by sequence homology analysis. In mice, Piezo1 and Piezo2 have high expressions in different MS tissues. For example, Piezo1 is abundant in MS cells in the skin, bladder, lung, kidney, and colon, whereas Piezo2 is highly expressed in dorsal root ganglia primary sensory neurons, and in the bladder, lung, and colon (Coste et al., 2010). Their subsequent study, also published by *Nature*, provides compelling evidence demonstrating that Piezo proteins, which have highly complex sequences and structures, are the first kind of pore-forming subunits of mechanically gated, non-selective cation channels in mammals (Coste et al., 2012). The importance of endothelial Piezo1 in vascular biology was first recognized from the finding by Li et al. (2014, *Nature*) that constitutive Piezo1 knockout (KO) in mice was embryonically lethal within days due to apparent defects in the developing vasculature (Li et al., 2014). The study by Woo et al. (2014, *Nature*) shows that Piezo2 is required for Merkel cell mechanotransduction and provides the first definitive evidence that Piezos play a physiological role in mechanosensation *in vivo.* Disruption of Piezo2 in the skin, but not in sensory neurons, resulted in reduced static firing rates in mice and decreased behavioral responses to gentle touch stimulation (Woo et al., 2014). In addition, based on the prior results, Ranade et al. (2014, *Nature*) precisely localized Piezo2 to the peripheral endings of a broad range of low-threshold mechanoreceptors. The results highlight that Piezo2, which displays rapidly adapting MA currents *in vitro*, is responsible for the mechanosensitivity of most low-threshold mechanoreceptor subtypes involved in innocuous touch sensation (Ranade et al., 2014b).

### Future Outlook

Our co-occurrence network maps, clustered by topic area or publication date, indicated the current hot topics and future directions in Piezo channel research (Figures 6A–C). The keywords indicated that Piezo channels are involved a variety of cell types (Merkel cells, neurons, endothelial cells, RBCs), physiological processes (touch sensation, blood pressure, proprioception, vascular development), related ion channels (TRP, Gardos), and diseases (pain, distal arthrogryposis, DHS, cancer), and pharmacology (Yoda1, GsMTx-4). The latest keywords that indicate future trends in this field are as follows.

**1. Architecture of Piezo Proteins.** Piezo proteins have a predicted sized of >2,500 amino acid residues and bear no resemblance to other proteins in this complex (Coste et al., 2010). Purified mouse Piezo1 is reconstituted into asymmetric lipid bilayers and liposomes to form ruthenium-red (RR)-sensitive ion channels (Coste et al., 2012). Researchers capitalizing on the technical breakthrough of cryo-electron microscopy (cryo-EM) have subsequently determined the 3D structure of Piezo proteins. Ge et al. contributed the first determination of the 3D cryo-EM structure of the recombinant full-length 2,547-residue mouse Piezo1 at a medium resolution. Piezo1 trimeric forms a remarkable three-bladed, propeller-like architecture, with three distal “blades” and a central “cap” on the extracellular side, and three distinct “beams” on the intracellular side (Ge et al., 2015). The higher-resolution structures of Piezo1 and Piezo2 have revealed the characteristics that may be related to mechanical gating machinery (Guo and MacKinnon, 2017; Saotome et al., 2018; Zhao et al., 2018; Wang et al., 2019). Although Piezo1 and Piezo2 share approximately only 42% sequence homology (Coste et al., 2010), both are homotrimer channels with similar propeller-shaped structures, which have a unique 38-transmembrane (TM) helix topology (Wang et al., 2019). Each blade consists of nine repetitive TM helical units (THUs), with each THU composed of fourTM segments. All nine THUs are solved in the Piezo2 structure (Wang et al., 2019). Three to six THUs closer to the central pore are assigned in the Piezo1 structure, but the three THUs at the peripheral region are currently too variable to be resolved by cryo-EM techniques (Guo and MacKinnon, 2017; Saotome et al., 2018; Zhao et al., 2018). The whole blade is connected to the central pore through an anchor domain. The central pore is formed by two TM α-helices (TM 37–38) adjacent to the C-terminal domain (CTD) (Jin et al., 2020). Charged amino acids located at the interface between the beam and the CTD facilitate the mechanical activation of Piezo2. Moreover, ensuring normal mechanosensitivity of Piezo2 requires hydrophobic interactions between the highly conserved Y2807 of the CTD and the pore-lining helices (Taberner et al., 2019). Understanding the gating process more thoroughly may require Piezo1 and Piezo2 structures in different functional states and other biophysical properties.

**2. Activation mechanism of Piezo Channels.** There are two basic principles of MS channel gating: force-from-lipids and force-from-filament (Cox et al., 2019). Piezo1 is gated through a force-from-lipid mechanism, suggesting that lipid tension alone is sufficient for gating Piezo1 (Syeda et al., 2016a; Cox et al., 2016). Considering the force-from-lipids paradigm of Piezo, lipids are crucial in the Piezo mechanosensing mechanism. Recent work from many labs is focusing on the role of specific lipids in Piezo channel activation. Saturated fatty acids can inhibit Piezo1 activation, whereas polyunsaturated fatty acids modulate channel inactivation and sensitize the Piezo1–GFP response to applied pressure (Romero et al., 2019; Ridone et al., 2020). The building evidence suggests that disrupting/depleting of cholesterol attenuates Piezo channel mechanosensitivity (Qi et al., 2015; Ridone et al., 2020). Ceramide and sphingomyelin are determinants of native Piezo gating that enable sustained activity (Shi et al., 2020). Piezo channel activity also requires the presence of phosphoinositides (Borbiro et al., 2015; Buyan et al., 2020; Jiang et al., 2021). Piezo1 and Piezo2 channels can locally deform lipid membranes into a dome-like shape (Zhao et al., 2018; Wang et al., 2019). In addition, changes in the projection area of Piezo channels from closed to open are essential for their mechanosensitivity (Guo and MacKinnon, 2017). The membrane dome mechanism has been proposed to explain the activation mechanisms of Piezo channels (Guo and MacKinnon, 2017; Lin et al., 2019). Xiao et al. proposed a lever-like mechanogating mechanism of Piezo1. The THU-constituted blade functions as the mechanosensing module, while the beam forms an effective lever-like apparatus for coupling the distal blade and the central pore module of Piezo1 (Wang et al., 2018; Zhao et al., 2019; Xiao, 2020). It has also been proposed that Piezo channels might utilize an elegant “plug-and-latch” mechanism to physically and coordinately gate the lateral portals by unplugging the plug gates (Geng et al., 2020).

**3. Pharmacology of Piezo Channels.** Although the current pharmacology of Piezo channels remains poor, there has been progress regarding small-molecule modulators of Piezo1. Yoda1 and Jedi1/2 are Piezo1 chemical activators that can activate Piezo1 channels in the absence of mechanical stimulation. Yoda1 was screened and identified from 3.25 million compounds via a cell-based fluorescence assay in 2015 (Syeda et al., 2015). Yoda1 activated purified Piezo1 in the absence of other cellular components, suggesting that it might directly act on Piezo1 (Syeda et al., 2015). Yoda1 binds Piezo1 to facilitate force-induced protein motions through a wedge-like mechanism, effectively lowering the channel’s mechanical threshold for activation (Botello-Smith et al., 2019). Jedi1/2, a novel set of Piezo1 chemical activators, activates Piezo1 through the peripheral blade. Jedi1/2 act on the upstream blade, while Yoda1 acts at the downstream beam. By utilizing Yoda1 and Jedi1/2, Xiao and collogues revealed that Piezo1 uses the peripheral blade-beam-constituted lever-like apparatus designated for long-distance mechanical and chemical gating of the pore (Wang et al., 2018). However, the chemical activators for the Piezo2 channel remain unclear.

RR and the peptide toxin GsMTx-4 are commonly used nonspecific blockers for Piezo1 and Piezo2 (Coste et al., 2010; Bae et al., 2011). RR, a polycation, blocks mouse Piezo1-and Piezo2-induced MA currents (Coste et al., 2010). Extracellular RR inhibits the inward but not outward current of mouse Piezo1/2, suggesting a pore-blocking mechanism (Coste et al., 2012). GsMTx-4 is an amphipathic peptide toxin widely used as an identifier and tool for investigating the physiological role of MA channels (Bowman et al., 2007). Both GsMTx4 enantiomers (L- and D-form) blocked Piezo1, inhibiting single-channel and whole-cell MA currents (Bae et al., 2011). GsMTx4 might not act directly on Piezo1 protein; rather, it relaxes the outer monolayer, thereby reducing the effective magnitude of stimulus acting on the Piezo1 gate (Gnanasambandam et al., 2017).

**4. Piezo1/2 in blood pressure regulation.** Both Piezo1 and Piezo2 have a role in the baroreceptor reflex that regulates blood pressure (Zeng et al., 2018). In mice, double KO of Piezo1 and Piezo2 in the nodose and petrosal sensory ganglia abolished drug-induced baroreflex and aortic depressor nerve activity, with hypertension and increased blood pressure variability. Optogenetic activation of Piezo2-positive sensory afferents was sufficient for initiating the baroreflex to decrease the heart rate and blood pressure in the mice. Considering the importance of the baroreceptor reflex in cardiovascular disease, targeting Piezo1 and Piezo2 might be a novel strategy for treating hypertension. However, the role of Piezos in the human baroreceptor reflex remains to be verified (Xiao, 2020).

**5. Relationship between Piezo1 and Gardos channel.** Piezo1 and the Gardos channel are both Ca^2+^ sensors in the RBC membrane, which control RBC volume. Gain-of-function mutations in Piezo1 have been associated with human DHS, characterized by severe dehydration, RBC atrophy, and anemia (Zarychanski et al., 2012; Albuisson et al., 2013). In addition, Piezo1 is required for shear-induced ATP release and calcium influx in human RBCs (Cinar et al., 2015). Piezo1 also plays a role in RBC metabolism. It increases the transmembrane flux of Ca^2+^ to stimulate Ca-ATPase and increases glycolysis in RBCs (Kuchel and Shishmarev, 2017; Kuchel et al., 2021).

The Gardos channel (also annotated as KCNN4, KCa3.1, IK1, or SK4) transfers Ca^2+^ uptake into K^+^ and water loss, thereby mediating Ca^2+^-dependent volume regulation and changes in RBC rheology (Kaestner et al., 2020). Mutations in the Gardos channel have also been linked to DHS (Andolfo et al., 2015; Glogowska et al., 2015; Rapetti-Mauss et al., 2015). Piezo1-mediated Ca^2+^ influx activates K^+^ efflux and subsequent dehydration of RBCs via downstream activation of the Gardos channel, directly indicating the relationship between Piezo1 and the Gardos channel in human RBC volume (Cahalan et al., 2015). Prolonged Gardos channel activation leads to changes in cell volume, which eventually causes other hemolytic diseases, such as sickle cell disease. The Gardos channel might be a pharmacological target for treating RBC diseases (Nagalla and Ballas, 2018).

**6. Role of Piezo Channels in Cancer.** Tumor cells are exposed to extracellular environments with mechanical stimuli, such as tissue pressure (stiffness), cell membrane tension, and ECM. Increased ECM stiffness, an important clinical tumor feature, activates MS channels to regulate tumor development (Fujii et al., 2020). In addition, Piezo channel activation can deliver local Ca^2+^ influx, thereby modulating key Ca^2+−^dependent signaling pathways associated with cancer cell migration, proliferation, and angiogenesis (De Felice and Alaimo, 2020). Recent studies have noted the close correlation between the Piezo1 channel and some cancers, including gastric cancer, oral squamous cell carcinoma, prostate cancer, colon cancer, synovial sarcoma, and osteosarcoma (Jiang et al., 2017; Suzuki et al., 2018; Zhang et al., 2018; Han et al., 2019; Sun et al., 2020; Hasegawa et al., 2021; Wang et al., 2021). Piezo1-dependent mechanosensation has also been linked to immune regulation in the differentiation of suppressive myeloid cells that regulate cancer and infectious diseases (Aykut et al., 2020). Furthermore, the Piezo2 channel plays a role in laryngeal squamous cell carcinoma, bladder cancer, and breast cancer (Cheng et al., 2017; Etem et al., 2018; Lou et al., 2019). Piezo channels might be a potential therapeutic target in cancer treatment.

**7. Role of Piezo Channels in Glaucoma.** Increased intraocular pressure (IOP) represents the most important risk factor for the onset and progression of glaucoma (Križaj, 2019). IOP reflects the balance between the production and outflow of aqueous humor. The trabecular meshwork, responsible for aqueous humor outflow and IOP maintenance, is very sensitive to mechanical forces. Piezo1 is widely expressed in the trabecular meshwork. Furthermore, Piezo1-derived MS currents have also been recorded in trabecular meshwork cells (Zhu et al., 2021). Piezo2 mRNA was expressed in the astrocytes of the optic nerve head, and its expression level was higher in glaucoma model mice (Choi et al., 2015). Recent findings have indicated that Piezo channels may be involved in the retinal ganglion cell (RGC) damage caused by high IOP. Both Piezo1 and Piezo2 were expressed in the corneal epithelium, lens epithelium, optic nerve head, and RGCs. Yoda 1 suppressed neurite outgrowth in RGCs, whereas, GsMTx4 promoted it (Morozumi et al., 2020). Further research on Piezo channels in the eye can clearly clarify the mechanisms in glaucomatous eyes.

### Strengths and Limitations

To the best of our knowledge, this is the first bibliometric analysis to investigate the research trend for Piezo channels. We conducted a comprehensive survey of the literature to perform quantitative and qualitative analyses of the publication output and quality of studies from different authors with the R bibliometrix package. We also used a well-known scientometric software tool (VOSviewer) to construct and visualize the bibliometric networks through co-authorship, co-citation, and co-occurrence analyses. Nevertheless, our analyses have some limitations. First, the searches were mainly conducted in the WoS database. It would be better to combine the results with that from other databases, such as PubMed and Scopus. However, it is worth noting that WoS is the most commonly used database in scientometrics and most bibliometric softwares could identify the format from WoS. Second, it appears that we only included English-language studies from WoS. Nevertheless, we performed the literature search without any language restriction. The articles included in WoS are provided with English abstracts for reading. Third, the keyword analysis results might have been affected by incomplete keyword extraction. For better visualization of keywords, keywords that occurred more than five times were presented in the network. We might have neglected the latest research trends because of the low occurrence of the related keywords. However, we additionally captured keywords from the publications in the recent two years. Accordingly, we discussed the role of Piezo channels in glaucoma. Fourth, as this is an emerging and developing research field, we might have underestimated the contribution to different analyses of recently published studies because of their low citation frequency, although some of the studies were published in high-quality journals.

## Conclusion

Our results indicate that the Unites States is a major contributor to Piezo channel research. Dr. Patapoutian of Scripps Research Institute is an outstanding scientist who has made a significant impact in this field. The majority of related studies are published in high-quality journals, suggesting that progress in this field is very meaningful. The keywords indicate that Piezo channels are involved a variety of cell types (Merkel cells, neurons, endothelial cells, RBCs), physiological processes (touch sensation, blood pressure, proprioception, vascular development), related ion channels (TRP, Gardos), and diseases (pain, distal arthrogryposis, DHS, cancer), and pharmacology (Yoda1, GsMTx-4). The focus has evolved from Piezo identification to architecture, activation mechanism, roles in diseases, and pharmacology.

## Data Availability

The original contributions presented in the study are included in the article/Supplementary Material, further inquiries can be directed to the corresponding authors.
